# Trendelenburg-Like Gait, Instability and Altered Step Patterns in a Mouse Model for Limb Girdle Muscular Dystrophy 2i

**DOI:** 10.1371/journal.pone.0161984

**Published:** 2016-09-14

**Authors:** Joseph W. Maricelli, Qi L. Lu, David C. Lin, Buel D. Rodgers

**Affiliations:** 1 School of Molecular Biology, Washington Center for Muscle Biology, Washington State University, Pullman, Washington, United States of America; 2 Department of Neurology, Carolinas Medical Center, Charlotte, North Carolina, United States of America; 3 Voiland School of Chemical Engineering and Bioengineering, Department of Integrative Physiology and Neuroscience, Washington Center for Muscle Biology, Washington State University, Pullman, Washington, United States of America; Rutgers University Newark, UNITED STATES

## Abstract

Limb-girdle muscular dystrophy type 2i (LGMD2i) affects thousands of lives with shortened life expectancy mainly due to cardiac and respiratory problems and difficulty with ambulation significantly compromising quality of life. Limited studies have noted impaired gait in patients and animal models of different muscular dystrophies, but not in animal models of LGMD2i. Our goal, therefore, was to quantify gait metrics in the fukutin-related protein P448L mutant (P448L) mouse, a recently developed model for LGMD2i. The Noldus CatWalk XT motion capture system was used to identify multiple gait impairments. An average galloping body speed of 35 cm/s for both P448L and C57BL/6 wild-type mice was maintained to ensure differences in gait were due only to strain physiology. Compared to wild-type mice, P448L mice reach maximum contact 10% faster and have 40% more paw surface area during stance. Additionally, force intensity at the time of maximum paw contact is roughly 2-fold higher in P448L mice. Paw swing time is reduced in P448L mice without changes in stride length as a faster swing speed compensates. Gait instability in P448L mice is indicated by 50% higher instances of 3 and 4 paw stance support and conversely, 2-fold fewer instances of single paw stance support and no instance of zero paw support. This leads to lower variation of normal step patterns used and a higher use of uncommon step patterns. Similar anomalies have also been noted in muscular dystrophy patients due to weakness in the hip abductor muscles, producing a Trendelenburg gait characterized by “waddling” and more pronounced shifts to the stance leg. Thus, gait of P448L mice replicates anomalies commonly seen in LGMD2i patients, which is not only potentially valuable for assessing drug efficacy in restoring movement biomechanics, but also for better understanding them.

## Introduction

Limb-girdle muscular dystrophies (LGMD) cause proximal muscle weakness around the hips and shoulders [1] and result not from x-linked mutations like the more common Duchenne muscular dystrophy, but from several dominant and recessive mutations that respectively produce LGMD1 and LGMD2 [2]. Of the 32 LGMD subsets, many result from mutations in proteins of the dystroglycan complex or in those that help stabilize the complex [3]. The latter include the putative glycotransferase fukutin-related protein (FKRP) [2, 4, 5], which is involved in glycosylation of α-dystroglycan as this is a prerequisite for binding to laminin [4–6]. In turn, mutations in FKRP compromise muscle cell binding to the extracellular matrix and produce a variety of pathologies with varying severity. This includes LGMD2i and the more severe Walker-Warburg Syndrome and Muscle-Eye-Brain Disease [7, 8].

Potential disease-causing mutations in FKRP occur in roughly 1 of 10,000 births and LGMD2i is often diagnosed in late adolescence or in adults [2]. Pathology presents similar to other LGMD subtypes, but is marked by highly elevated serum creatine kinase (CK) levels. Dilated cardiomyopathy and respiratory complications are also common and are the underlying cause of mortality [1, 7–13]. There is currently no cure for LGMD2i as research has been hampered by the relatively low disease prevalence and lack of appropriate animal models, at least until recently. The transgenic FKRP P448L knock-in mouse [7] possesses a proline-to-leucine missense mutation at residue 448, which is the most common mutation seen in human LGMD2i patients. Moreover, initial studies of this model suggest that it recapitulates many disease markers exhibited by human patients including elevated serum CK, stereotypic histological markers of muscle degeneration and muscle weakness [7, 8, 11, 14]. Phenotypic expression of pathology typically first presents at 2 or 3 weeks of age as the hind legs retract and in rare occurrences, more severe disorders of eye and brain developmental have been noted [7]. Another LGMD2i mouse model expressing a milder phenotype, the FKRP L276I mutant mouse [8, 15], has also been developed. Replacing wild-type FKRP in these mice restores muscle structure and some aspects of function [16]. However, no whole animal assessments of muscle function or exercise have been performed on this or any mouse model of LGMD2i before or after FKRP replacement.

Gait assessment studies in human patients with any form of muscular dystrophy are limited and studies with animal models are even rarer [17–21]. The primary gait metric used clinically with muscular dystrophy patients is the 6-minute walk test and this is used to determine mobility rather than locomotion patterns [22, 23]. Because FKRP mutations produce varying levels of pathology with both early and late onset, loss of mobility can occur anywhere between 11 and 60 years of age [2]. A “waddling” gait is common among LGMD patients [24] due to muscle weakness in the hip and pelvic girdle, although it is not known whether this or any other gait metric predicts loss of mobility, which is among the earliest and often most life-altering symptom in many muscular dystrophy patients. This myopathic waddling gait, called Trendelenburg gait, is consistent with other muscular dystrophies that exhibit hip abductor weakness and body weights shifts to the support leg in stance [25–27]. Thus, a better understanding of gait in muscular dystrophy patients and in dystrophic animal models could not only help to understand how and why mobility impairment develops, from a biomechanics perspective, but also to identify systemic markers of disease pathology that could be used in both pre-clinical and clinical drug trials.

Reported herein is the first quantitative assessment of gait in any LGMD2i animal model. Our results identified many similarities between P448L mice and human LGMD patients including a “waddling gait” where the hips appear to drop and most or the entirety of the paws were touching the surface during maximum contact creating a shuffling appearance (supplemental video 1). P448L mice also require more support from other legs while galloping, which limits step patterns and promotes a radial “giraffe walk” or pacing pattern. This gait substantially differs from that of wild-type mice and provides multiple metrics for assessing drug efficacy in this important animal model and possibly in other dystrophic mice or even muscular dystrophy patients.

## Materials and Methods

### Animal Care

Experiments were performed in male and female wild-type C57Bl/6 and P448L congenic mice, which also possess a C57Bl/6 genetic background, bred and housed in an IACUC accredited facility at Washington State University. Animals were fed and watered *ad libitum* and housed in an environmentally controlled room under a 12 h light/dark cycle. All handling and experimental protocols were preapproved by an Institutional Animal Care and Use Committee in accordance with the Guide for the Care and Use of Laboratory Animals. Two month old mice were used as this is a common age used in drug and gene therapeutic studies with other dystrophic lines and because at this age, both wild-type and P448L mice have similar body weights and lengths. Although hydrocephaly and other developmental defects were previously noted in P448L mice, none of these defects was detected in any of the mice used in this study.

### Catwalk Setup

Six male and female mice of each genotype were run through the Catwalk XT system (Noldus Information Technology, Wageningen, NL) that has a camera mounted underneath a glass walkway to capture individual print, footfall and force profiles measured using a light intensity equivalent as described [28]. The camera was set 9 inches away from the walkway before capture and the green light threshold that captures print outlines and intensities on the walkway was set between 0.10 and 1.00 with a frame capture rate of 100/sec. Mice were coaxed to run by gently tapping on the walkway or blowing and runs longer than 5 seconds and shorter than 1.5 seconds were automatically excluded in order to maintain consistent speeds. Furthermore, each mouse was assessed individually for 3 consecutive viable runs and those that resulted in speed variance exceeding 20% were also excluded. Mice completed 3–4 step cycles in frame per run allowing the system to create average run statistics from the 3 individual runs. Each of these run averages was then used to create mean statistics between the mouse models.

### Gait Cycle

**Fig 1:**
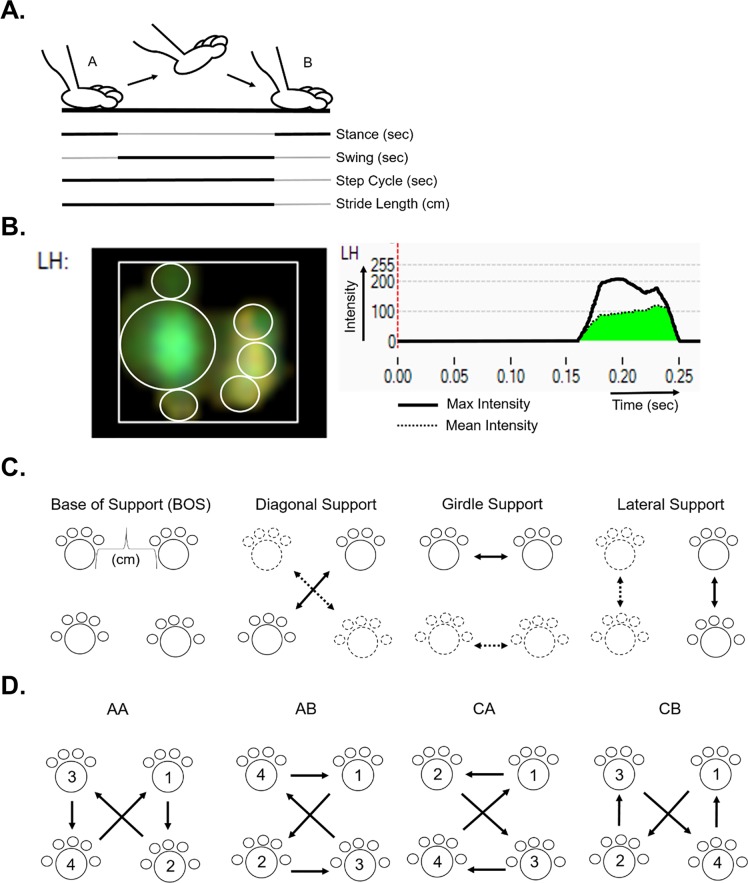
Gait metric overview. (A) Single paw representation of gait cycle between point A and B along a walkway. Separate gait metrics are defined by solid black lines within the gait cycle. (B) Screen capture images of the Noldus Catwalk System software showing print measurements and recorded intensities for the left hind paw of a P448L mouse. Light displacement leaves a green paw print on the walkway as shown and is used to derive print surface area metrics and intensity. The latter is an indication of weight supported by the paw. Intensity is measured in real time, graphed and used to calculate the highest intensity pixel in the print, Max Intensity (black line), and the Mean Intensity of all pixels (green area under curve). (C) Summary of support metrics. Solid prints and arrows represent a support pair, the opposite pairs are represented as fragmented prints and arrows. (D) Common step patterns include the alternate patterns AA and AB and the cruciate patterns CA and CB. Numbers and arrows indicate step order. The right front paw was arbitrarily chosen as the initial step.

The Catwalk system provides several measurement variables automatically (indicated by italics in the text). *Swing time* is the time a paw is airborne from one footfall to the next and *stride length* is the distance covered per swing. Dividing stride length by swing time results in the *average swing speed* of the paw. The *step cycle* is the time it takes to complete a single cycle of a step, effectively the sum of stance and swing time. The *duty cycle* then explains what percentage of the whole step cycle is encompassed by the stance phase:

dutycycle=(stand/stepcycle)X100%

where *stance* (referred to as stand by the equipment) is the duration of the stance phase and *step cycle* is the duration of stance and swing phases combined (Fig 1A).

### Paw Contact

The stance phase is the time from initial to final paw contact where the transition between braking and propulsion occurs. During stance, the paw contact length, width and area are recorded over the entire stance phase as *print length*, *width*, and *area*, respectively, and at *maximum contact area* (Fig 1A and 1B). *Stand index* is the speed at which the paw leaves the walkway. It is measured using the following equation where “a” is the slope of the line and “X0” is the camera frame rate when the paw is at max contact:

StandIndex=(a/X0)−framerate

*Time to max contact* measures the time maximum contact is recorded relative to the beginning of the stance phase and is determined by the following equation:

Timetomaxcontact=(MaxcontactAt–Initialcontact)/StandX100%

where *Max contact At* is the time of stance phase when the maximum foot area is in contact with the glass, *Initial contact* is the start time of the stand phase (when the foot first touches the walkway) and *Stand* is the stance time duration.

### Paw Intensity

In addition to measuring timing and paw metrics during the stance phase, the Catwalk system also quantifies step intensities based on the amount of scattered light within a scale of 0 to 255. Intensity measurements are reported at specific instances, such as at maximum contact or are integrated throughout the stance period. These intensities reflect weight over the paws when they are in contact with the walkway, which the machine correlates to the force exerted by the paws. *Max contact max intensity* measures the mean of the highest intensity pixel of the print image at the maximum print contact for each step. *Max contact mean intensity* is the mean for all pixels in the print image during maximum print contact (Fig 1B). *Max intensity* refers to the mean of the highest recorded pixel intensity through the entire stance phase while *time to max intensity* (referenced as *max intensity at* by Noldus) quantifies the time required to reach *max intensity*.

### Support

Support from each limb in contact with the surface, while other limbs are in swing, can be measured during each step cycle and as a percentage of the whole recorded run. *Initial* and *terminal dual stance* measure the amount of time that both contralateral paws are on the walkway at the start and end of the step cycle, respectively. The distance between contralateral paws during *initial* and *terminal stance* is referred to as *base of support* (*BOS*). *Single stance* refers to the time a paw in swing is supported by the contralateral paw. The percent of support throughout the run can be parsed into the total number of paws as well as special instances with only 2-paw support (i.e. diagonal, contralateral, and girdle) (Fig 1C).

### Step Patterns

Quadrupeds can potentially use multiple step sequence patterns when galloping and walking [29]. The Catwalk system records the actual order of footfalls as they occur and categorizes them based on common and uncommon sequence patterns. The *number of different patterns* used and the *regularity index* reflect the percentage of defined gait patterns used during the run according to:

regularityindex=(normalsteppatterns*4)/pawplacementX100%

where *normal step patterns* is the total amount the defined AA, AB, CA, or CB gait patterns (see below) were used and *paw placement* is the total paw placement patterns recorded. The frequency of the four most common quadruped step patterns can then be determined and include the following: (AA) RF-RH-LF-LH, (AB) LF-RH-RF-LH, (CA) RF-LF-RH-LH and (CB) LF-RF-LH-RH and can be visualized in the summary figure (Fig 1D).

### Statistical Analysis

Statistical analyses were performed using Prism (GraphPad Software; La Jolla, CA). Initial tests assessed sex as an independent variable and identified no effect of sex in any parameter. The results from both sexes were, therefore, combined (n = 12) for each group and significant differences between means (-/+ SEM) were determined using a student’s t-test (p≤0.05).

## Results

### Body Speed and Variance

As speed is an underlying factor that affects gait, maintaining body speed and its variation between mouse groups was critical. Body speeds were maintained at a gallop with no significant differences between groups (wild-type: front paws 35.45±8.80 cm/s, hind paws 35.77±8.03 cm/s; P448L: front paws 33.99±3.89 cm/s, hind paws 34.37±4.65 cm/s) [30, 31]. Additionally, there were no differences in body weights between the two groups independent of sex (data not shown). These results indicate that all proceeding differences in gait are due to gait mechanics and not speed.

### Gait Cycle

**Fig 2:**
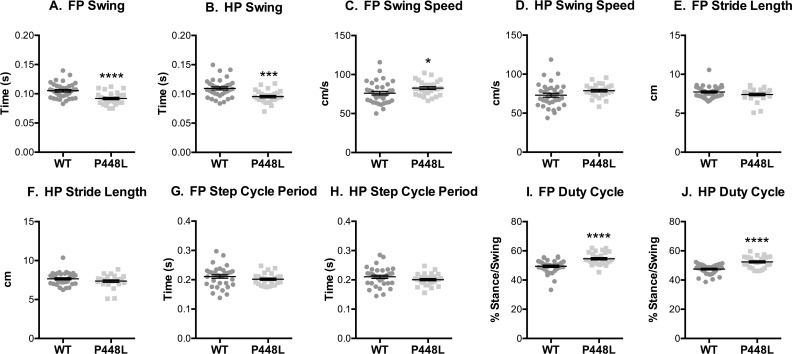
Gait Cycle. (A,B) Fore paw and hindpaw measurements of the time paws are airborne and (C,D) the speed at which paws are moving. (E,F) The total distance travelled by the paw during swing. (G,H) The time it takes from initial paw contact with the ground from one step to the initial paw contact of the preceding step. (I,J) The proportion of time that the paw spends on the ground compared to in the air. (FP = fore paw, HP = hindpaw, n = 12; *p<0.05; ***p≤0.001, ****p≤0.0001).

*Swing time* is less in P448L mice, indicating that they spend significantly less time with their paws in the air and this is consistent with a higher forepaw swing speed (Fig 2A–2D). However, these differences did not lead to longer a *stride length* or slower *step cycle* (Fig 2E–2H). This suggests that although the velocity and cadence between the groups were similar, the stance phase in the P448L must be longer relative to swing as this compensates for less swing time. This is confirmed in the *duty cycle* measurement as P448L mice proportionally spend more time in the stance phase compared to swing phase (Fig 2I and 2J). This is not to say that stance time is significantly different between mouse groups, but the percentage of the step cycle spent in the stance phase is higher.

### Paw Contact

**Fig 3:**
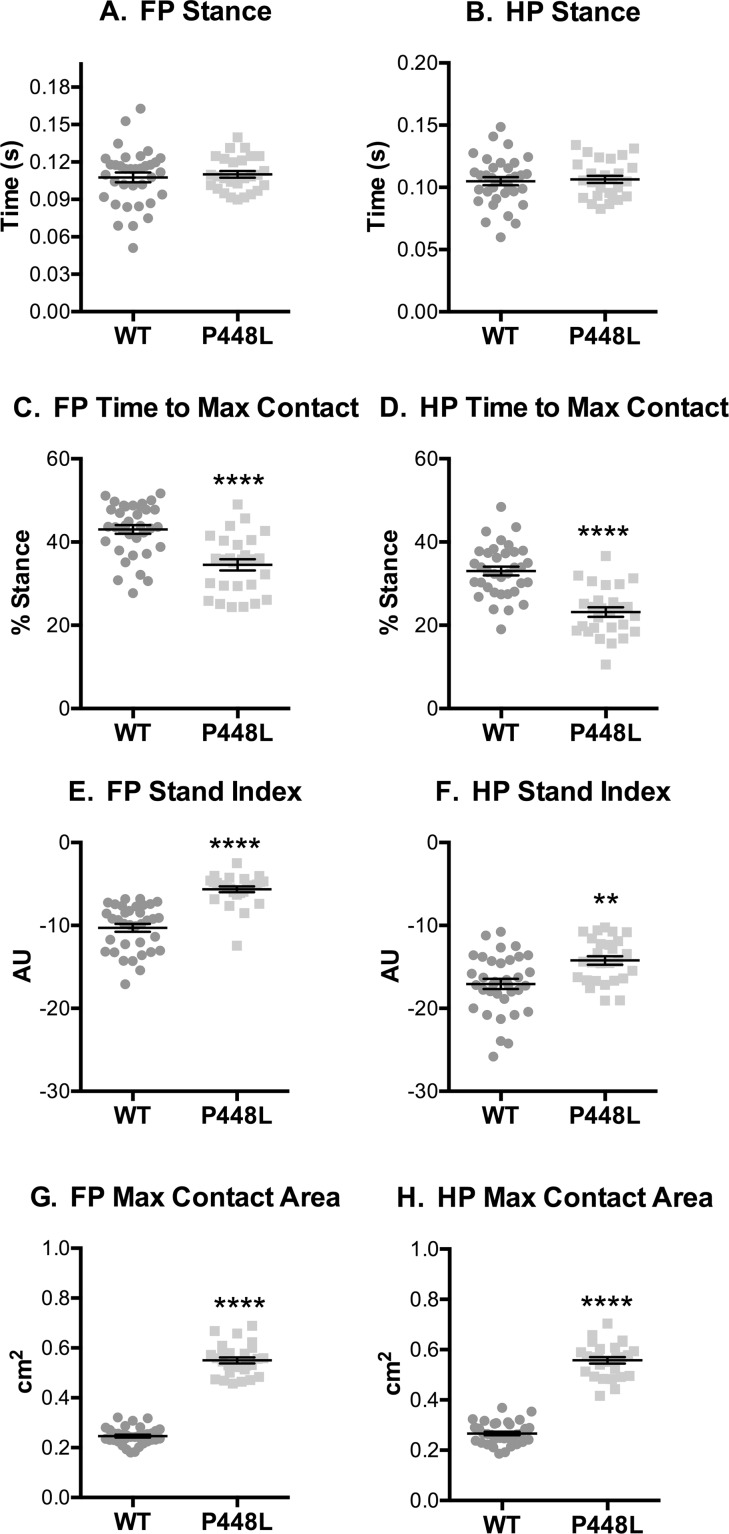
Paw Contact Times. (A,B) Fore paw and hindpaw measures of the time from initial ground contact to the paw becoming airborne for a single step. (C,D) The time it takes from initial contact with the walkway to the highest paw surface area with the ground represented as the percentage of the Stance Time that Maximum Contact was reached. (E,F) The speed of the paw leaving the walkway (AU, arbitrary units derived as “a” from y = ax+b, a best fit line; see methods). (G,H) Area of the paw at the time of maximum contact. (FP = fore paw, HP = hindpaw, n = 12; **p≤0.01, ****p≤0.0001).

**Fig 4:**
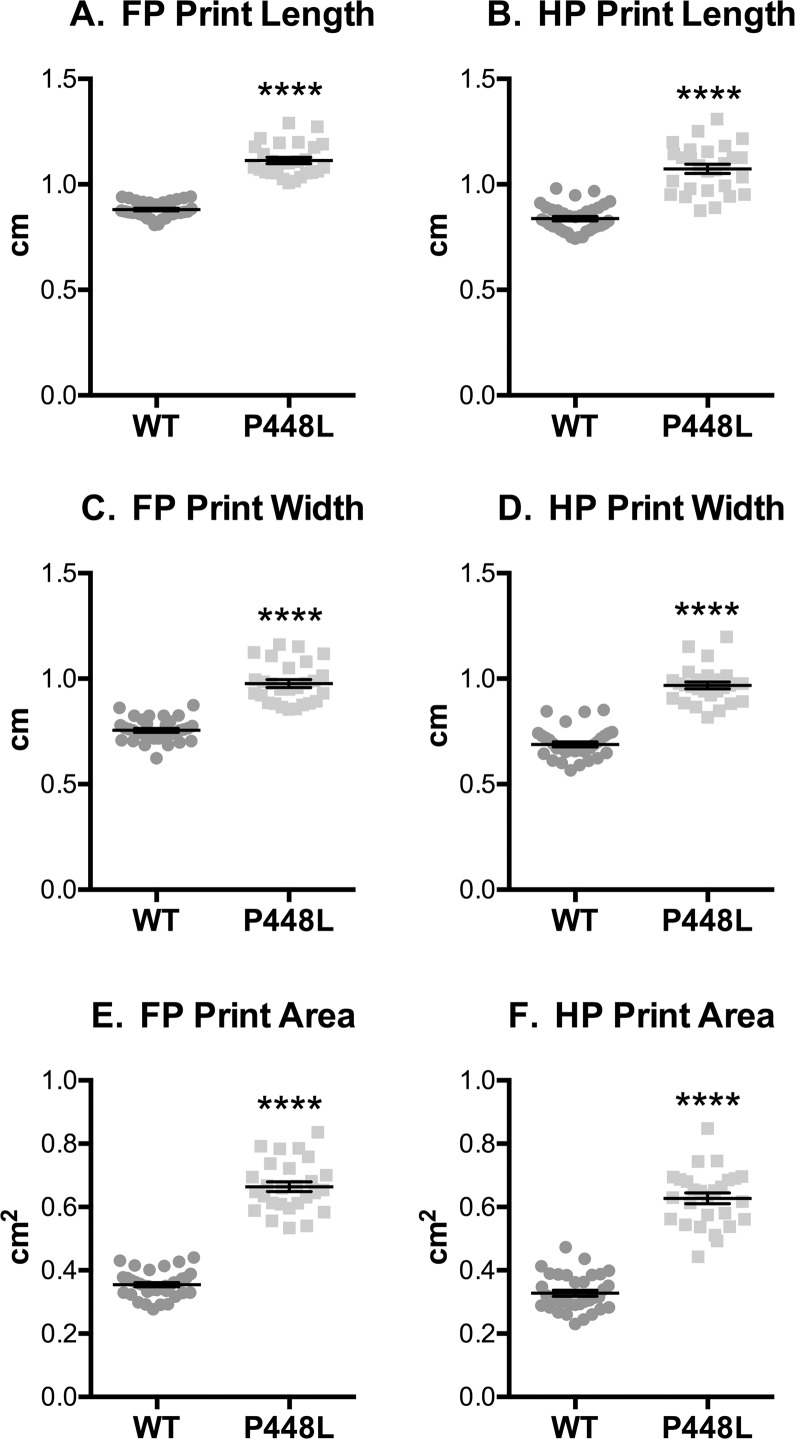
Paw Contact/Print Area. Fore paw and hindpaw measurements of print length (A,B), width (C,D) and area (E,F) recorded from the entire stance phase. (FP = fore paw, HP = hindpaw, n = 12; ****p≤0.0001).

The stance phase of the step cycle begins when paws contact the walkway and ends when paws leave and initiate swing. Although *stance* was similar in both groups (Fig 3A and 3B), the time at which paws come into maximal contact with the walkway, which represents the transitionary phase between braking and propulsion, is reached in P448L mice roughly 10% faster than wild-type (Fig 3C and 3D). This in turn lead to a faster speed of the paws leaving the walkway for P448L mice as indicated by a larger *stand index* (Fig 3E and 3F). The total surface contact area of the paws was 2.5 times larger in P448L mice (Fig 3G and 3H). Indeed, *print length* and *width* throughout the entire stance phase are roughly 30% larger in both front and hind paws of P448L mice, resulting in 70% larger total print area (Fig 4A and 4F). This indicates that P448L mice gallop with a more “foot slap” gait, which could account for the shortened braking period and the need for a longer propulsion phase during stand (Fig 3C and 3D).

### Paw Intensities

**Fig 5:**
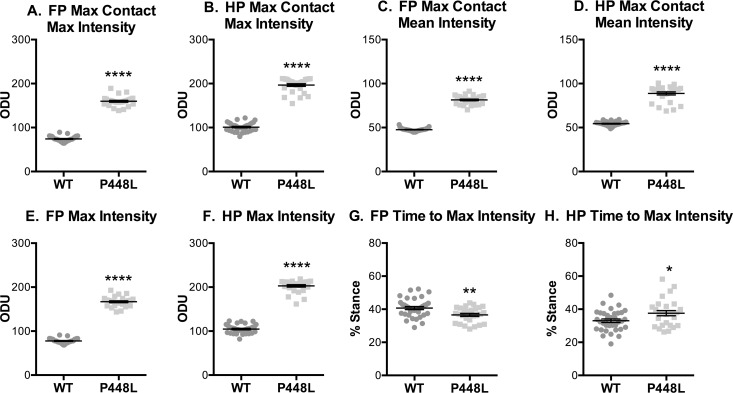
Paw Intensities. Fore paw and hindpaw measurements of greenlight intensity with an optical density green light threshold of 0 to 255. These measurements include (A,B) the highest and (C,D) average pixel intensity during the time of maximum contact with the walkway. (E,F) This also includes the highest intensity recorded at any point during stance phase and (G,H) the time it takes to reach the highest intensity as represented as a percentage of the stance time. (FP = fore paw, HP = hindpaw, ODU = Optical Density Units, n = 12; *p<0.05; **p≤0.01, ****p≤0.0001).

The step weight placed on the paws by P448L mice, as indicated by the intensity of light scatter, was significantly higher than that of wild-type regardless of metric. Maximum and mean intensities at the time of maximum contact were 2-fold higher (Fig 5A–5D), consistent with the maximum intensities of the whole paw throughout the stance phase (Fig 5E and 5F). This is likely due to a higher shift in weight between paws during stance and an extended propulsion phase in P448L mice. *Time to max intensity* refers to the time it takes paws to reach the largest weight exerted on the walkway in proportion to the stance phase, much like the *time to max contact* metric. These two percentiles are normally identical or very similar, as seen with the wild-type paws. However, *time to max contact* (Fig 3C and 3D) and *time to max intensity* (Fig 5G and 5H) are uncoupled in P448L hind paws. The P448L hind paws exhibit a disconnect between *max contact* and *max intensity*, with the *max contact* reached at 23% of the total stance phase as opposed to 37% for the *max intensity*. Thus, the mice are braking during the stance phase with a larger weight being placed on the walkway and braking faster than wild-type in their hind legs. This means that proportionally more weight is being used to stop momentum in the P448L mice than during the beginning of the propulsion phase, which transitions roughly at the point of max contact as seen in the wild type mice. This is again consistent with a foot slap gait, but additionally suggests that gait pathologies are more apparent in P448L hind paws.

### Stance and Support

**Fig 6:**
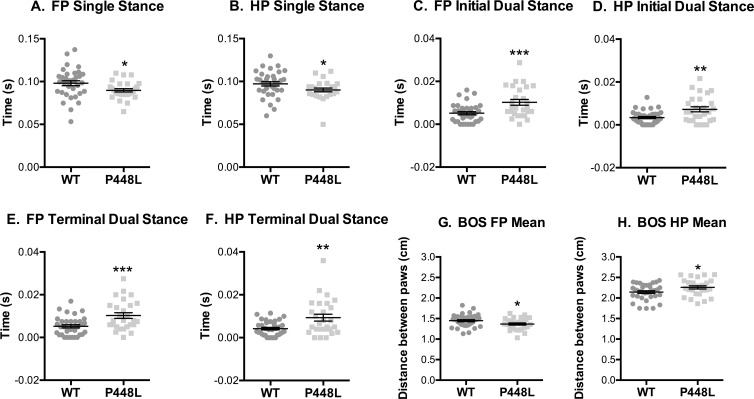
Stance and Contralateral Paws. (A,B) Fore paw and hindpaw measurement of ground contact duration for a single paw while the contralateral paw is airborne. (C,D) The time contralateral paws simultaneously contact the ground at the beginning of a step and (E,F) at the end of a step. (G,H) The distance between contralateral paws while both are in stance phase. (FP = fore paw, HP = hindpaw, n = 12; *p<0.05; **p≤0.01, ***p≤0.001).

Step cycle differences in the support by contralateral limbs suggest that P448L mice require more support when running. The time spent with one paw in swing and the contralateral paw on the surface (single stance) was less in P448L mice (Fig 6A and 6B). Conversely, initial and terminal dual stance measures in P448L mice, measures of simultaneous contralateral support, were double those of wild-type (Fig 6C–6F). These data partially explain why swing time is shorter in P448L mice, which compensate their instability by rapidly accelerating limbs through the step cycle in order to provide greater support with more paw contacts. The base of support in hind paws was larger in P448L mice, although forepaw support was smaller (Fig 6G and 6H). This is not particularly surprising as hind paws carry more proportional mass than forepaws.

**Fig 7:**
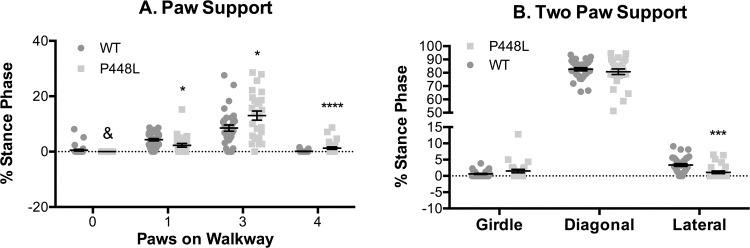
Paw Support. (A) Percentage of run duration that no paws, a single paw, three paws or four paws were simultaneously touching the surface. (B) Percentage of run duration that two diagonal paws (RF-LH or LF-RH), contralateral paws (girdle, RF-LF or RH-LH) or lateral paws (RF-RH or LF-LH) were simultaneously touching. Students t-test was performed between WT and P448L in each group and combined into a single panel. (n = 12; *p<0.05; **p≤0.01, ***p≤0.001, ****p≤0.0001 & denotes 0 recorded instances).

Similar differences were also detected when support metrics assessed all paws over the course of the entire run. At no time did P448L mice gallop with all paws in the air and their relative time with only one paw on the surface was approximately half that of wild-type mice (Fig 7A), which is consistent with lower single stance measures (Fig 6A and 6B). By contrast, the relative time with 3 or 4 paws on the surface was 63% and 125-fold higher, respectively, in P448L mice (Fig 7A), and this is again consistent with higher dual stance measures (Fig 6C–6F). Multi-paw support in P448L mice was not distributed equally as the frequency of diagonal support (e.g. front left and right hind) was similar in both groups (Fig 7B). The frequency of girdle support (both paws on a side), however, tended to be higher in P448L mice and although this difference was not significant, the frequency of lateral support was less than a third of the wild-type percentage (Fig 7B). The latter results do not conflict with a higher incidence of dual stance in P448L mice as lateral support metrics are collected when only two paws touch the surface and P448L mice gallop predominantly with three or even four paws touching.

### Step Patterns

**Fig 8:**
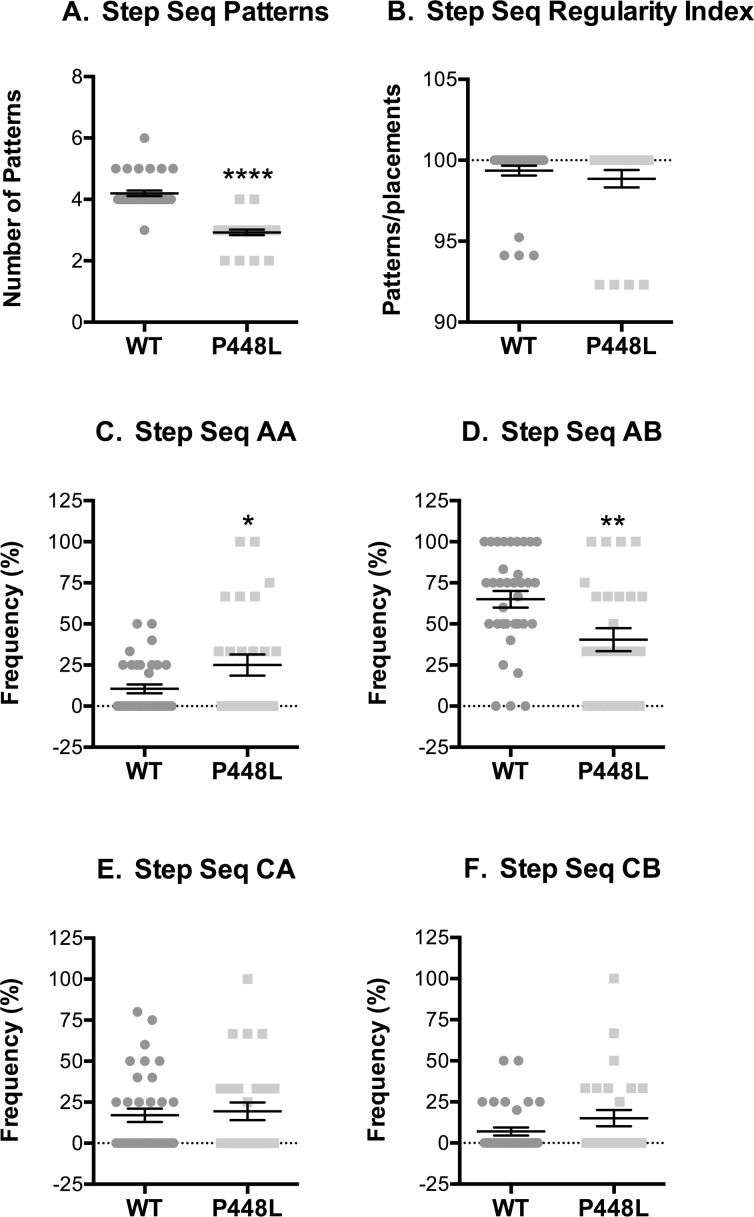
Step sequence. (A) Number of distinct step sequence patterns used. (B) The percentage of recognizable normal patterns used during a run (R, right; L, left, F, forepaw; H, hind paw; normal patterns = AA, RF-RH-LF-LH; AB, LF-RH-RF-LH; CA, RF-LF-RH-LH; CB, LF-RF-LH-RH). (C-F) Frequency of use for the indicated patterns. (n = 12; *p<0.05; **p≤0.01, ***p≤0.001).

P448L mice used fewer step sequence patterns than wild-type mice, although the *regularity index* was similar in both groups indicating that they predominantly used the four most common patterns (Fig 8A and 8B). The primary difference in patterns used by wild-type and P448L mice were in the frequency of radial patterns. P448L mice used the AA “giraffe walk” pattern 2.5-fold more often than the wild-type mice that used, as is typical, the AB pattern 40% more often than did P448L mice (Fig 8C and 8D). Alternating contralateral step CA/CB patterns were used at the same frequency between strains and at smaller frequency than the radial AA/AB patterns (Fig 8E and 8F). Thus, the varied gait mechanics and need for greater support in P448L mice is accompanied by the use of fewer, albeit common, step sequence patterns and a reliance on the AA radial gait pattern that would result in a similarly higher use of girdle support.

## Discussion

**Fig 9:**
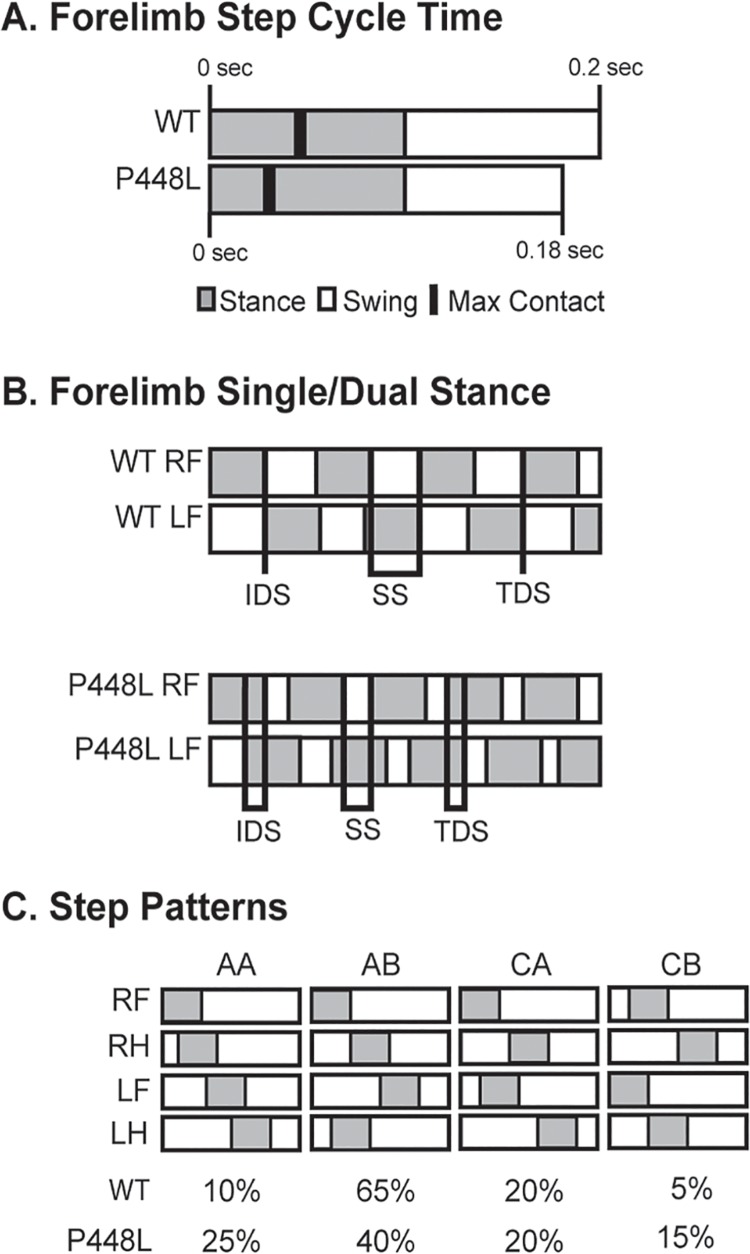
Gait summary. (A) Visual representation of the step cycle over time in wild-type (WT) and P448L mice. Each bar signifies a single step cycle lasting the indicated time. Stand phase represents a greater proportion of the cycle, maximum contact is reached earlier and swing time is less in P448L mice while stride length is unaffected due to compensatory faster swing speeds. (B) Visual representation of differences in initial dual stance (IDS), single stance (SS), and terminal dual stance (TDS) between WT and P448L mice (gray boxes = paw in contact with surface; lines = periods of IDS, SS or TDS). The longer IDS and TDS and shorter SS in P448L mice reflect longer periods of contralateral paws touching the walkway during the gait cycle and, conversely, less time with only one contralateral paw touching. This was also reflected in greater instances of 3 and 4 paw support in P448L mice. (C) Hildebrand plot of the common gait patterns: AA (RF-RH-LF-LH), AB (LF-RH-RF-LH), CA (RF-LF-RH-LH) and CB (LF-RF-LH-RH). Percentages indicate relative use of each step pattern in each mouse group. The most common step pattern in both was the alternating AB pattern, although P448L mice used it significantly less and compensated with an increase use of the radial AA pattern.

Understanding the adverse effects on gait in a disease state that causes loss of ambulation has the potential to influence patient management and even the development of novel treatment strategies and therapeutics. This highlights the importance of our studies as they are the first to quantify abnormal gait patterns in any dystroglycanopathy model, regardless of specific disease type, and are by far the most comprehensive assessment of gait in any dystrophic animal model to date. Compared to wild-type mice, the “waddling” or Trendelenburg-like gait of P448L mice is exemplified by a lesser time to maximal paw contact, greater paw surface contact, higher paw intensity measures, smaller paw swing times and the requirement of greater limb support, which is consistent with a higher frequency of irregular step patterns and pacing (summarized in Fig 9A–9C). These results correspond with some human muscular dystrophy gait metrics [22, 32–36] and despite the obvious differences in bipedal versus quadruped gait, they illustrate the translational utility of assessing gait in P448L mice. Indeed, these studies identified several novel biomechanical disease markers that would facilitate the testing of drug candidates and other therapeutic interventions in whole animals.

The distinctive P448L gait is readily distinguishable (see S1 Movie of mice on a treadmill). It is also consistent with a Trendelenburg gait [26, 27, 37] that commonly develops with different muscular dystrophies and myopathies and results from weakened muscles of the hip abductors as in LGMD2i patients [2, 24, 38]. This causes the hips to dip excessively as subjects walk and often results in a characteristic “waddle” and “foot slap” [39–41], which also occur in P448L mice. Although the P448L hip abductors have yet to be functionally analyzed, hind limb retraction time is compromised in 2 week old P448L mice as is hind limb grip strength [7, 15], all of which is consistent with limb and hip muscle dysfunction.

The Trendelenburg-like gait in conjunction with flat step patterns partly explain why paw intensity metrics are higher in P448L mice. These metrics reflect paw intensities generated during the step cycle and although it seems counterintuitive that dystrophic animals generate more force with each step, this could be explained by a less fluid gait pattern causing hard footfalls (i.e. foot slap) that require higher weight shifts during a faster braking period and, therefore, an extended propulsion period. This is supported by the fact that max intensity in P448L mice is reached before max contact and this coincides with higher instability and need for more support (i.e. swing time is lower). However, intensity only truly measures proportional weight exerted on the walkways and does not provide a quantitative measure of actual exerted force, which is a limitation of the measure.

It is logical to assume that a Trendelenburg-like gait in a quadruped might require heightened support from contralateral limbs. This indeed occurs in P448L mice and coincides with a faster swing speed and less swing time (Fig 2). In fact, a slower swing time results from added stress on the hip abductors in patients with a bilateral Trendelenburg gait [42]. The P448L mice can further compensate weakened limbs by using multi-paw support with three or more paws touching the platform simultaneously, more time in dual stance of contralateral paws and a wider hind limb base of support (Figs 4, 6 and 7). Step patterns are also significantly affected as P448L mice rely heavily on just a few patterns, especially radial step patterns that are uncommon in wild-type mice (Fig 9). One shortcoming in the Catwalk system is that it does not measure the joint kinematics that can also influence gait mechanics [43, 44]. Such technologies have recently been developed for hindlimb assessments and could potentially be used to confirm the Trendelenburg-like gait and especially the kinematics of pelvic tilt [45]. It is also possible that problems in neural developmental may have contributed to differences in gait mechanics as FKRP is expressed in muscle and brain. We suspect this is highly unlikely as none of the mice used in this study displayed the previously reported neural abnormalities, in fact none have occurred in our breeding colony, and more importantly, the Trendelenburg gait also occurs in human patients with other forms of muscular dystrophy. The phenotype described is, therefore, a consequence of muscle weakness rather than neural deficits.

Early diagnosis of muscular dystrophies allows for earlier preventative treatment and extends patient ambulatory times especially as minor gait issues are often among the first noticed symptoms [22, 32, 46]. This underscores the need to perform gait analyses in other muscular dystrophy animal models and in human patients as such studies, especially in mice, are extremely rare. The use of gene therapeutics to treat various forms of muscular dystrophy has shown significant promise. This includes studies using *mdx* mice to replace mutated dystrophin with microdystrophin and those using L276I mice to restore wild-type FKRP [16, 47–51]. The Catwalk system provides an opportunity to assess these and other dystrophic mice *in vivo* using a “real world” metric–gait–which our studies demonstrate is similarly affected in P448L mice and human LGMD2i subjects. A better mechanistic understanding of gait abnormalities in P448L mice could, therefore, help to better understand the biomechanical nature of similar pathologies in humans and possibly more important, to aid in the screening of novel drugs and or gene therapeutics for treating LGMD2i.

## Supporting Information

S1 Movie Treadmill Running Gait. C57BL/6 wild-type and P448L mice were recorded running on the Columbus Instruments Oxymax FAST treadmill system. The treadmills were set at 12 m/min and the video was slowed to 60% speed.
(MOV)
